# Vitamin D levels and platelet status in healthy children and adolescent

**DOI:** 10.3389/fnut.2025.1682472

**Published:** 2025-11-26

**Authors:** Pedro Antonio Souza de Almeida, Polyana Romano Oliosa, Leticia Batista de Azevedo, José Geraldo Mill, Miriam Carmo Rodrigues Barbosa

**Affiliations:** 1 Postgraduate Program in Nutrition and Health, Federal University of Espírito Santo (UFES), Vitória, Brazil; 2 Postgraduate Program in Public Health, Federal University of Espírito Santo (UFES), Vitória, Brazil; 3 Postgraduate Program of Physiological Sciences, Federal University of Espírito Santo (UFES), Vitória, Brazil

**Keywords:** vitamin D, blood platelets, child, adolescents, cardiovascular disease

## Abstract

**Background:**

Recent studies have expanded our understanding of the functions of vitamin D beyond bone health and calcium homeostasis. Limited data exists on the prevalence of vitamin D insufficiency in Brazil, particularly in children, but studies have shown deficiencies in a significant percentage of the population. Some studies have suggested a potential interaction between vitamin D and platelets, as they share common metabolic pathways, and alterations in platelet levels have been observed in individuals with vitamin D insufficiency.

**Objective:**

This study aimed to verify if serum levels of 25(OH)D, platelet count, and mean platelet volume (MPV), are associated in children and adolescents aged 6 to 17 years old.

**Methods:**

This is a cross-sectional study in children and adolescents aged 6 to 17 years (first sentence) Serra, Espírito Santo, a metropolitan city in Southeast Brazil. In total, 659 children and adolescents aged 6 to 17 years old and registered in a social project called “Estação do Conhecimento” in Serra, Espírito Santo, Brazil. Socioeconomic data were collected in the first interview using a semi-structured questionnaire. Blood was collected after 12 h of fasting, 25-hydroxyvitamin D [25(OH)D] levels were assessed using a chemiluminescence method, blood cells were analyzed using the counting method by impedance, and the cell analysis were performed by continuous flow cytometry.

**Results:**

Vitamin D insufficiency was highly prevalent among children and adolescents. Vitamin D levels and platelets were positively correlated and independently associated. Surprisingly, insufficient and deficient vitamin D were associated with higher mean platelet volume.

**Conclusion:**

Our findings suggest that vitamin D’s influence on platelet parameters is likely indirect, possibly mediated by its role in modulating low-grade inflammation.

## 1 Introduction

Vitamin D, traditionally associated with bone metabolism and calcium homeostasis, has gained increasing attention for its pleiotropic effects, particularly its involvement in immune regulation, cardiometabolic processes, and inflammatory (1, 2). Recent evidence has linked hypovitaminosis D to increased cardiovascular risk, including associations with hypertension, metabolic syndrome, and endothelial dysfunction in both adults and pediatric populations (2–4).

Despite Brazil’s tropical climate, vitamin D insufficiency is prevalent among children and adolescents, often attributed to urban lifestyle, reduced sun exposure, and increasing rates of childhood obesity (5). This has raised public health concerns regarding its potential role in the early development of atherothrombotic disease, especially as platelet activation and volume—represented by mean platelet volume (MPV)—have been recognized as early biomarkers of vascular inflammation and thrombosis.

Platelets are not only key players in hemostasis but also participate in immune signaling and inflammatory cascades. MPV is considered a dynamic indicator of platelet activation and has been associated with cardiovascular risk in both adults and children (6, 7). Previous studies investigating the relationship between vitamin D status and platelet indices have yielded conflicting results, often focusing on adult populations or patients with comorbidities, such as type 2 diabetes, immune thrombocytopenia, or multisystem inflammatory syndrome in children (6–8).

Previous investigations into the relationship between vitamin D status and platelet indices have yielded conflicting results and have predominantly focused on adult populations or children with existing comorbidities (6–8). Consequently, there is a significant gap in the literature concerning this association in healthy pediatric cohorts. Establishing this baseline relationship is essential for understanding early cardiovascular risk markers without the confounding influence of underlying disease states.

This study aims to evaluate the relationship between serum 25-hydroxyvitamin D [25(OH)D] concentrations and platelet indices, including platelet count and MPV, in a large sample of healthy Brazilian children and adolescents. By focusing exclusively on a non-pathological population, we seek to contribute novel evidence on the underlying physiological relationship between vitamin D status and platelet behavior in youth.

## 2 Materials and methods

### 2.1 Study design

This was a cross-sectional study conducted in Serra, Espírito Santo, Brazil. Participants were children and adolescents enrolled in the “Estação do Conhecimento,” a non-profit community program funded by the Vale Foundation. The program provides educational support, music instruction, and sports activities for school-aged youth from low-income neighborhoods.

### 2.2 Study setting and period

The study was carried out in Serra, a metropolitan municipality in the state of Espírito Santo. The Estação do Conhecimento, established in 2011, partners with public and private institutions to provide an extensive social support network. It serves children as young as 6 years old, offering structured after-school programming, and includes income-generating activities for older adults. Data collection occurred between 2017 to 2020.

### 2.3 Study population and eligibility criteria

Approximately 1,100 children and adolescents aged 6–18 years were invited to participate in a study examining cardiovascular parameters across different ancestry groups. Inclusion criteria were enrollment in the program, informed consent provided by parents or guardians, and assent from adolescents aged 12 and above. Data was collected from June 2018 to November 2019 in a non-probabilistic population, and 671 children and adolescents completed all the stages of the study. For the present study, participants who did not have biochemical data were excluded.

### 2.4 Data collection

Demographic and clinical data were collected by trained interviewers using structured questionnaires. Demographic variables included age (years), sex, and self-reported race/ethnicity (White, Black, or Multiracial). Pubertal stage was assessed according to Tanner criteria and categorized as pre-pubertal, pubertal, or post-pubertal. Additionally, parents or legal guardians provided the child’s medical history and information on current medication usage.

### 2.5 BMI

Fasting blood samples were collected via venipuncture in the early morning and analyzed by a centralized certified laboratory using standardized procedures. For this study, platelet count was analyzed as both a continuous variable (x10^3^/μL) and a categorical variable (thrombocytopenia, defined as <150 ×10^3^/μL) (9). Additionally, mean platelet volume (fL) was included as an analytical parameter. Serum 25-hydroxyvitamin D [25(OH)D] levels were measured using chemiluminescence with the ARCHITECT® platform (Abbott Diagnostics, Lake Forest, IL, USA). Results were expressed in ng/mL and classified into two groups: inadequate (< 20 ng/mL) and adequate (≥ 20 ng/mL) (10). The complete blood count was performed using impedance and continuous flow cytometry (BECKMAN COULTER DxH800®).

### 2.6 Statistical analysis

The Kolmogorov–Smirnov test was initially used to assess data normality, and the results indicated a normal distribution, allowing all analyses to be conducted using appropriate statistical tests. Continuous variables are presented as mean ± standard deviation; categorical variables are presented as frequencies and percentages. To compare characteristics between groups with adequate and inadequate Vitamin D status, the independent samples t-test and the chi-square test were used. Effect sizes were calculated using Cohen’s d and Cramér’s V, respectively. Pearson’s correlation was used to assess the linear association between continuous variables.

To evaluate the independent association between 25(OH)D levels and platelet count, a multiple linear regression model was constructed, adjusting for age, sex, and BMI. Regression results are presented as unstandardized coefficients (B) with their corresponding 95% confidence intervals (95% CI). All analyses were performed using SPSS (version 21.0; SPSS Inc., Chicago, IL, USA), with statistical significance set at *p* < 0.05.

### 2.7 Ethical considerations

The study protocol was approved by the Human Research Ethics Committee at the Health Sciences Center of the Federal University of Espírito Santo (CAAE: 30385014.8.0000.5060, protocol no. 725.488). All participants and their guardians were informed about the study objectives and procedures. Written informed consent was obtained from guardians, and assent was provided by adolescents aged 12–18 years in accordance with ethical standards.

## 3 Results

The study included a total of 659 children and adolescents with a mean age of 11.1 ± 2.8 years. Most participants were female (58.7%), and the majority were classified in the pubertal stage (59.8%). Vitamin D deficiency was observed in 71 participants, representing 10.8% of the total sample.

The demographic and clinical characteristics of the population, stratified by vitamin D status, are detailed in Table 1. The group with insufficient vitamin D presented a significantly higher mean age and BMI in comparison to the group with adequate levels of vitamin D (*p* < 0.001; ES = 0.51 and *p* = 0.031; ES = 0.27, respectively). Additionally, statistically significant associations were observed between vitamin D status and the variables sex (*p* = 0.026; ES = 0.08), and prevalence of thrombocytopenia (*p* = 0.011; 0.09).

**Table 1:** Demographic and health characteristics of the sample, according to the 25(OH)D concentration.

Variables	Total (*N* = 659)	Vitamin D adequate (*N* = 588)	Vitamin D inadequate (*N* = 71)	*p*	Effect size
Age, years, Mean ± S.D	11.1 ± 2.7	10.9 ± 2.7	12.4 ± 2.8	<0.001*	0.51
BMI, kg/m^2^, Mean ± S.D	19.2 ± 5.2	20.5 ± 4.6	19.1 ± 5.2	0.031*	0.27
Sex, *n* (%)				0.026**	0.08
Masculine		234 (39.8)	38 (53.5)		
Feminine		354 (60.2)	33 (46.5)		
Race/ethnicity, *n* (%)				0.177**	0.07
White		166 (28.2)	21 (29.6)		
Black		170 (28.9)	27 (38.0)		
Multiracial		252 (42.9)	23 (32.4)		
Platelets, *n* (%)				0.011**	0.09
Adequate		586 (99.7)	69 (97.2)		
Thrombocytopenia		2 (0.3)	2 (2.8)		

BMI, body mass index. **Chi-square test (ES calculated by Cramér’s V). *Student *t*-test (ES calculated by Cohen’s d).

Table 2 shows the comparison between groups with adequate and inadequate vitamin D levels. The group with inadequate vitamin D presented a significantly higher MPV compared to the group with adequate levels (9.35 fL vs. 9.11 fL; *p* = 0.035; ES = 0.26). The platelet count was, on average, lower in the group with vitamin D inadequacy (268.41 ×10^3^/μL) in relation to the adequate group (281.48 ×10^3^/μL); however, this difference did not reach statistical significance (*p* = 0.118; ES = 0.19) (see Table 3).

When stratified by sex, correlation analyses revealed a positive association between serum vitamin D levels and platelet count (*r* = 0.119, *p* = 0.049 M; *r* = 0.102, *p* = 0.045 F). Conversely, a negative correlation was observed between vitamin D levels and MPV (*r* = −0.119, *p* = 0.049 M; *r* = −0.105, *p* = 0.038 F).

**Table 2:** Comparison between groups with adequate and inadequate vitamin D levels.

Variables	Vitamin D adequate (*N* = 588)	Vitamin D inadequate (*N* = 71)	*p*	Effect size
Platelet count, 10^3^/μL, Mean ± S.D	281.48 ± 66.66	268.41 ± 66.48	0.118	0.19
Mean platelet volume, fL, Mean ± S.D	9.11 ± 0.38	9.35 ± 1.05	0.035	0.26

*T*-Student test (effect size calculated by Cohen’s d).

**Table 3:** Platelet count and volume according to plasma 25(OH)D levels in children and adolescents, stratified by sex.

Variables	Vitamin D, ng/mL			
	Male		Female	
	*r*	*p*	*r*	*p*
Platelet count, 10^3^/μL, Mean ± S.D	0.119	0.049	0.101	0.045
Mean platelet volume, %, Mean ± S.D	−0.119	0.049	−0.105	0.038
Pearson’s correlation.				

When stratified by sex, correlation analyses revealed a positive association between serum vitamin D levels and platelet count (*r* = 0.119, *p* = 0.049 M; *r* = 0.102, *p* = 0.045 F). Conversely, a negative correlation was observed between vitamin D levels and MPV (*r* = −0.119, *p* = 0.049 M; *r* = −0.105, *p* = 0.038 F).

To evaluate the independent association of vitamin D with platelet parameters, multiple linear regression models were constructed, adjusted for age, sex, and BMI. In the adjusted model for platelet count (Table 4), vitamin D remained a positive and statistically significant predictor. Specifically, each 1 ng/mL increase in vitamin D levels was associated with an average increase of 0.53 ×10^3^/μL in platelet count (95% CI: 0.2, 1.1). For MPV, it was observed that each 1 ng/mL increase in vitamin D was associated with a reduction of 0.08 fL in MPV. However, this association showed only a trend, not reaching statistical significance in the adjusted model (95% CI: −0.1, 0.1) (see Table 5).

**Table 4:** Linear regression analysis of the association between 25(OH)D levels and platelet count.

Variables	Model 1		Model 2	
	*β* (95%*CI*)	*p*	*β* (95%*CI*)	*p*
Vitamin D, ng/mL	0.72 (0.2, 1.2)	0.008	0.53 (0.2, 1.1)	0.043

B, beta; CI, confidence interval. Model 1 was crude model. Model 2 was adjusted for age, sex, and body mass index.

**Table 5:** Linear regression analysis of the association between 25(OH)D levels and mean platelet volume.

Variables	Model 1		Model 2	
	*β* (95%*CI*)	*p*	*β* (95%*CI*)	*p*
Vitamin D, ng/mL	−0.11 (−0.1, −0.1)	0.009	−0.08 (−0.1, 0.1)	0.051

B, beta; CI, confidence interval. Model 1 was crude model. Model 2 was adjusted for age, sex, and body mass index.

## 4 Discussion

Our main finding demonstrates that, even after adjusting for potential demographic and anthropometric confounders, higher 25(OH)D levels remained significantly associated with a higher platelet count. Concomitantly, an inverse relationship was observed with MPV, a marker of young and reactive platelets, although this association showed borderline statistical significance in the multivariate model. Collectively, this hematological profile, characterized by a reduced platelet count and an elevated MPV in states of vitamin D insufficiency, suggests a state of accelerated platelet turnover. Our findings align with a recent study by Okuyan et al. (11) in children and adolescents demonstrated that vitamin D deficiency was associated with higher levels of inflammatory markers, such as the Platelet-to-Lymphocyte Ratio (PLR), and with insulin resistance, reinforcing the hypothesis that vitamin D status modulates systemic inflammation and metabolism. Additionally, in a large retrospective cohort of over 16,000 children (12) also found statistically significant, yet weak, correlations between vitamin D deficiency and several hemogram-derived inflammatory markers, including PLR and Platelet Distribution Width. The authors suggest that the clinical significance of such weak associations should be interpreted cautiously, as statistical significance may be driven by the large sample size. This observation supports that vitamin D’s influence on platelet parameters is likely indirect and subtle, rather than a clinical effect of large magnitude.

A higher frequency of hypovitaminosis D was observed among male participants, which contrasts with much of the existing literature, where females typically present lower vitamin D levels (13, 14). This discrepancy may reflect population-specific differences such as body composition and behavioral factors; for instance, obese male adolescents may have reduced vitamin D bioavailability due to increased adiposity (15).

The positive correlation between vitamin D and platelet count remained statistically significant when stratified by sex. However, these findings contrast with studies in adult populations. For example, Park et al. (8) reported a weak but inverse correlation between 25(OH)D and platelet indices in Korean adults. A critical distinction lies in the study populations: while Park et al. included adults with varied health statuses, our study focused exclusively on healthy children and adolescents. The divergent results may thus reflect age-related physiological differences, varying hormonal profiles, or the absence of chronic inflammatory states in our cohort. Moreover, platelet dynamics and vitamin D metabolism are known to be modulated by sex hormones such as estrogen, further contributing to population-specific variability (16, 17).

The multivariate analysis showed that the association between vitamin D and platelet parameters persists even after adjusting for key confounders. Our results demonstrate that higher 25(OH)D levels were independently associated with an increased platelet count. Conversely, the inverse association with MPV, while suggestive of a profile of lower platelet turnover with vitamin D sufficiency, showed only a borderline trend in the fully adjusted model. This hematological profile suggests that the influence of vitamin D on platelet status is not direct, but rather mediated by broader systemic pathways, primarily inflammation (1, 17–19). Mechanistically, vitamin D may exert its effects on platelet behavior indirectly through its role in modulating inflammation and immune signaling. Calcitriol—the active form of vitamin D—can suppress pro-inflammatory pathways by inhibiting NF-κB activation and reducing cytokines such as TNF-α and IL-6 (1, 19). These cytokines are known to enhance megakaryopoiesis and promote the production of larger, more reactive platelets, leading to elevated MPV and increased thrombotic potential.

An additional hypothesis involves the role of cholesterol sulfate (Ch-S), a molecule secreted by red blood cells and platelets that maintains electrostatic repulsion and prevents thrombus formation. Ch-S synthesis is mediated by the sulfotransferase enzyme SULT2B1b, whose expression may be induced by vitamin D receptor (VDR) activation (20). Although this mechanism has been described primarily *in vitro*, it presents a plausible pathway by which vitamin D may influence platelet aggregation through electrochemical interactions (21).

In summary, our findings suggest that vitamin D does not exert a direct effect on platelet indices but may influence platelet physiology through inflammatory and metabolic pathways. The observed correlations, although statistically significant, are weak, which supports the view that vitamin D’s relationship with platelet parameters is mediated by broader immunological and endocrine mechanisms. In a pediatric population, a chronic low-grade inflammatory state driven by hypovitaminosis D could have significant long-term implications for the development of cardiovascular risk.

The use of platelet parameters in our study is situated within a broader and growing field of research seeking to validate novel, cost-effective hematological indices as markers of inflammation and prognosis in cardiovascular disease (22–24). For instance, a recent study by Eyiol et al. (22) demonstrated that new hemogram-derived ratios, such as the Hemoglobin-to-RDW Ratio and the RDW-to-Albumin Ratio, are valuable biomarkers for assessing severity and recurrence risk in patients with acute pericarditis. Although the specific markers and population differ, the underlying principle is the same: to extract maximal prognostic information from a routine blood test. In this context, our findings contribute to this field by suggesting that vitamin D status may modulate accessible platelet parameters in a pediatric population, highlighting the potential of these indices for subclinical risk stratification from an early age.

The public health implications of our findings, despite the weak correlations, relate to primordial prevention. It is suggested that the observed platelet alterations do not represent an immediate thrombotic risk, but rather serve as a subclinical biomarker of a low-grade inflammatory state associated with vitamin D insufficiency (1, 19). The identification of a pro-inflammatory state in youth is relevant, considering that pathological processes such as atherosclerosis can begin at an early age (4). Therefore, our results suggest that monitoring and maintaining vitamin D sufficiency in pediatric populations can be considered not only for bone health but also as a strategy to mitigate chronic inflammation and long-term cardiovascular risk (2, 4). This is a hypothesis that requires confirmation through longitudinal studies.

The primary limitations of this study include its cross-sectional design, which precludes causal inference, and the lack of direct measurement of inflammatory cytokines, which would help clarify the hypothesized indirect mechanisms. Additionally, residual confounding may exist due to unmeasured variables such as pubertal and socioeconomic status, and seasonality of blood collection, which were not fully accounted for. Also, the mechanistic interpretation is speculative, as no inflammatory biomarkers were measured. However, the present study has several strengths. First, our analysis was conducted in a large and well-characterized cohort, exceeding the sample size of most previous investigations on this topic. Second, the focus on apparently healthy children and adolescents minimizes the potential for confounding by comorbidities, allowing for a clearer assessment of the association between vitamin D and platelet parameters.

## 5 Final considerations

This study identified a weak yet positive correlation between 25(OH)D levels and platelet count, as well as higher mean platelet volume in groups with insufficient vitamin D. Although this direction contrasts with some findings in adult populations—which report a negative correlation—the weak association in both contexts suggests a more nuanced and possibly indirect or population-specific relationship between vitamin D status and platelet indices.

Crucially, our findings support the hypothesis that the observed platelet alterations are not directly mediated by serum 25(OH)D levels per se but rather stem from the metabolic and inflammatory environment induced by vitamin D insufficiency. This context promotes low-grade systemic inflammation, which may contribute to increased platelet activation and the production of larger, immature platelets, thereby explaining the subtle shifts in MPV.

An additional contribution of this study is the suggestion of a possible indirect mechanism—via inflammatory pathways—that may link vitamin D deficiency to changes in platelet activity. This interpretation aligns with broader research suggesting that vitamin D modulates immune and inflammatory responses, which in turn influence thrombopoiesis and platelet behavior.

To move beyond correlational evidence and deepen our understanding of the vitamin D–platelet axis, future studies must adopt more sophisticated, multifaceted designs. These should include longitudinal and interventional frameworks to establish temporal and causal links, as well as molecular-level investigations to unravel the underlying mechanisms. Integrating direct measurements of inflammatory mediators, comprehensive platelet phenotyping, and potentially omics-based approaches (e.g., transcriptomics or proteomics) will be essential to reveal how vitamin D status impacts platelet function at the cellular and biochemical levels.

In conclusion, while the association between vitamin D and platelet indices remains modest, the proposed indirect mechanism via inflammation offers a compelling explanation for our findings and a promising avenue for future research into cardiometabolic health in pediatric populations.

## Data Availability

The raw data supporting the conclusions of this article will be made available by the authors, without undue reservation.

## References

[1] Martens P-J Gysemans C Verstuyf A Mathieu C. Vitamin d’s effect on immune function. Nutrients. (2020) 12:1248. doi: 10.3390/nu12051248, PMID: 32353972 PMC7281985

[2] Jorge AJL Cordeiro JR Rosa MLG Bianchi DBC. Vitamin D deficiency and cardiovascular diseases. Int J Cardiovasc Sci. (2018) 31:422–432. doi: 10.5935/2359-4802.20180025

[3] Pilz S Tomaschitz A. Role of vitamin D in arterial hypertension. Expert Rev Cardiovasc Ther. (2010) 8:1599–608. doi: 10.1586/erc.10.142, PMID: 21090935

[4] Xu W-R Jin H-F Du J-B. Vitamin D and cardiovascular risk in children. Chin Med J. (2017) 130:2857–62. doi: 10.4103/0366-6999.215500, PMID: 28948941 PMC5717866

[5] Stagi S Cavalli L Iurato C Seminara S Brandi ML de Martino M. Bone metabolism in children and adolescents: main characteristics of the determinants of peak bone mass. Clin Cases Miner Bone Metab. (2013) 10:172–9. 24554926 PMC3917578

[6] Feketea G Vlacha V Pop RM Bocsan IC Stanciu LA Buzoianu AD . Relationship between vitamin D level and platelet parameters in children with viral respiratory infections. Front Pediatr. (2022) 10:824959. doi: 10.3389/fped.2022.824959, PMID: 35463888 PMC9021877

[7] Cure MC Cure E Yuce S TarkanYazici Karakoyun I Efe H. Mean platelet volume and vitamin D level. Ann Lab Med. (2014) 34:98–103. doi: 10.3343/alm.2014.34.2.98 24624344 PMC3948840

[8] Park YC Kim J Seo MS Hong SW Cho ES Kim J-K. Inverse relationship between vitamin D levels and platelet indices in Korean adults. Hematology. (2017) 22:623–9. doi: 10.1080/10245332.2017.1318334, PMID: 28486836

[9] van Ommen CH Peters M. The bleeding child. Part I: primary hemostatic disorders. Eur J Pediatr. (2012) 171:1–10. doi: 10.1007/s00431-011-1532-4, PMID: 21800040 PMC3249149

[10] Ferreira CES Maeda SS Batista MC Lazaretti-Castro M Vasconcellos LS Madeira M . Consensus – reference ranges of vitamin D [25(OH)D] from the Brazilian medical societies. Brazilian Society of Clinical Pathology/laboratory medicine (SBPC/ML) and Brazilian Society of Endocrinology and Metabolism (SBEM). J Bras Patol Med Lab. (2017) 53:377–81. doi: 10.5935/1676-2444.20170060

[11] Okuyan O Dumur S Elgormus N Uzun H. The relationship between vitamin D, inflammatory markers, and insulin resistance in children. Nutrients. (2024) 16:3005. doi: 10.3390/nu16173005, PMID: 39275320 PMC11396811

[12] Konuksever D Yücel Karakaya SP Bölük O Koçak M Orhan Kılıç B Ünsal Saç R. The association of vitamin D deficiency with hemogram-derived inflammatory biomarkers in children. Nutr Metab Cardiovasc Dis. (2022) 32:2418–23. doi: 10.1016/j.numecd.2022.07.012, PMID: 35973886

[13] Wierzbicka A Oczkowicz M. Sex differences in vitamin D metabolism, serum levels and action. Br J Nutr. (2022) 128:2115–30. doi: 10.1017/S0007114522000149, PMID: 35042577

[14] David GG Maia JEF Freitas OCSD Caldeira AP. Prevalência e fatores associados à deficiência e insuficiência de vitamina D em adolescentes no norte de Minas Gerais. Rev Bras Saude Mater Infant. (2024) 24:e20240020. doi: 10.1590/1806-9304202400000020

[15] Turer CB Lin H Flores G. Prevalence of vitamin D deficiency among overweight and obese US children. Pediatrics. (2013) 131:e152–61. doi: 10.1542/peds.2012-1711, PMID: 23266927

[16] Kebapcilar AG Kulaksizoglu M Ipekci SH Korkmaz H Kebapcilar L Akyurek F . Relationship between mean platelet volume and low-grade systemic coagulation with vitamin D deficiency in primary ovarian insufficiency. Arch Gynecol Obstet. (2013) 288:207–12. doi: 10.1007/s00404-013-2735-x 23377179

[17] Dupuis M Severin S Noirrit-Esclassan E Arnal J-F Payrastre B Valéra M-C. Effects of Estrogens on platelets and megakaryocytes. Int J Mol Sci. (2019) 20:3111. doi: 10.3390/ijms20123111, PMID: 31242705 PMC6627332

[18] Coşkun C Şahin K. Correlation between vitamin D level and platelet indices in children aged 0-18 years. Mede Bull Haseki. (2018) 56:153–7. doi: 10.4274/haseki.41736, PMID: 31893583

[19] Wimalawansa SJ. Vitamin D deficiency: effects on oxidative stress, epigenetics, gene regulation, and aging. Biology (Basel). (2019) 8:30. doi: 10.3390/biology8020030, PMID: 31083546 PMC6627346

[20] Seo Y-K Mirkheshti N Song CS Kim S Dodds S Ahn SC . SULT2B1b sulfotransferase: induction by vitamin D receptor and reduced expression in prostate Cancer. Mol Endocrinol. (2013) 27:925–39. doi: 10.1210/me.2012-1369, PMID: 23579488 PMC3656233

[21] Cooper ID Crofts CAP DiNicolantonio JJ Malhotra A Elliott B Kyriakidou Y . Relationships between hyperinsulinaemia, magnesium, vitamin D, thrombosis and COVID-19: rationale for clinical management. Open Heart. (2020) 7:e001356. doi: 10.1136/openhrt-2020-001356, PMID: 32938758 PMC7496570

[22] Eyiol H Eyiol A Sahin AT. Clinical relevance of HRR (hemoglobin to RDW) and RAR (RDW to albumin) in pericarditis. Biomark Med. (2025) 19:197–204. doi: 10.1080/17520363.2025.2471743, PMID: 40013601 PMC11916353

[23] Nascimento MAL Ferreira LGR Alves TVG Rios DRA. Inflammatory hematological indices, cardiovascular disease and mortality: a narrative review. Arq Bras Cardiol. (2024) 121:e20230752. doi: 10.36660/abc.20230752, PMID: 39193999 PMC11495820

[24] Hu B Liu T Sun Y Sun J Feng L Li F. Association between systemic immune-inflammation index and all-cause and CVD mortality in non-elderly diabetic adults. Clinics. (2025) 80:100739. doi: 10.1016/j.clinsp.2025.100739, PMID: 40774029 PMC12347989

